# The Effect of Phosphorus Concentration on the Co-Production of Fucoxanthin and Fatty Acids in *Conticribra weissflogii*

**DOI:** 10.3390/md22120541

**Published:** 2024-11-30

**Authors:** Ning Zhang, Di Peng, Xiangyu Rui, Wenquan Zheng, Zhenglin Zeng, Xianghu Huang, Changling Li, Feng Li

**Affiliations:** College of Fisheries, Guangdong Ocean University, Zhanjiang 524088, China; zhangn@gdou.edu.cn (N.Z.); 2112201054@stu.gdou.edu.cn (D.P.); 2112001098@stu.gdou.edu.cn (X.R.); 11135432oo@stu.gdou.edu.cn (W.Z.); 202111111102@stu.gdou.edu.cn (Z.Z.); huangxh@gdou.edu.cn (X.H.); licl@gdou.edu.cn (C.L.)

**Keywords:** *Conticribra weissflogii*, phosphorus, fucoxanthin, fatty acids, co-production

## Abstract

The production of fucoxanthin and fatty acids in *Conticribra weissflogii* has been examined, but the role of elements like phosphorus in their mutualistic interactions is not well understood. To fill this gap, our study utilized potassium dihydrogen phosphate (KH_2_PO_4_) as a source of phosphorus to examine its impact on the synthesis of fucoxanthin and fatty acids in *C. weissflogii*. Our findings revealed that at a phosphorus concentration of 10 mg L^−1^, the cell density (9.5 × 10^5^ cells mL^−1^), carotenoid concentration (1.67 mg g^−1^), fucoxanthin concentration (0.91 mg L^−1^), and fucoxanthin content (1.33 mg g^−1^) were maximized. Additionally, at a phosphorus concentration of 20 mg L^−1^, cell dry weight (0.76 ± 0.08 g L^−1^), total fatty acid content, saturated fatty acids, and unsaturated fatty acids were all at their highest levels, making this concentration optimal for EPA accumulation. In conclusion, manipulating the phosphorus concentration can enhance the levels of fucoxanthin and unsaturated fatty acids in *C. weissflogii*, offering valuable insights into the co-production of these two high-value compounds within this species.

## 1. Introduction

Microalgae, as the primary carbon producers in marine ecosystems, fix carbon through photosynthesis and produce a variety of compounds with high diversity and bioactivity, making them one of the richest and most economical sources of natural compounds [1,2,3]. They are key nutrient carriers, introducing these essential nutritional compounds into the food chain. Microalgae are rich in nutrients and bioactive substances such as proteins, polysaccharides, lipids, and polyunsaturated fatty acids [4,5]. The bioactive substances in microalgae possess antioxidant, anti-inflammatory, anticancer, and antibacterial properties, making them highly valuable in the fields of medicine, cosmetics, and the chemical industry [6].

Fucoxanthin, a natural carotenoid, is a unique photosynthetic pigment found in microalgae and brown algae, playing a crucial role in photosynthesis [7]. Its unique chemical structure and rich bioactivity provide high commercial value, making it widely used in cosmetics, health products, and pharmaceuticals [8]. Research has shown that compared to the high cost and unsustainability of large brown algae, many unicellular microalgae have high fucoxanthin content, fast growth rates, low extraction costs, and short cultivation cycles, making them important alternative sources for commercial fucoxanthin production [9,10]. However, while many scholars have studied the synthesis of fucoxanthin and fatty acids, the impact of phosphorus on their synthesis remains unclear.

In the natural environment, carotenoids serve as ubiquitous biologically active substances that offer numerous benefits to organisms and are recognized for their display of red, yellow, and orange hues [11]. These compounds originate from the biosynthetic processes of various photosynthetic organisms (including plants, algae, and cyanobacteria) as well as some non-photosynthetic organisms. Microalgae represent a rich source of carotenoids, exhibiting immense potential for production. Carotenoids in microalgae generate energy through a process similar to photosynthesis in higher plants, utilizing pyruvate and glyceraldehyde 3-phosphate as raw materials to synthesize the common precursor of all known carotenoids, isopentenyl diphosphate (IPP), and its isomer dimethylallyl diphosphate (DMAPP), via the 2-methyl-D-erythritol-4-phosphate (MEP) pathway [12]. The carotenoids produced by microalgae are predominantly composed of 9-cis and all-trans isomers. Compared to chemically synthesized carotenoids, they possess advantages such as naturalness, high efficiency, and ease of industrial production, thus holding significant scientific research value and commercial potential [13]. Research on the production of carotenoids using microalgae is abundant, with *Dunaliella salina*, one of the prime sources of β-carotene, and *Haematococcus pluvialis*, a major source of astaxanthin, being the key algal species currently used for the large-scale production of carotenoids [14].

Diatoms are not only known for their rich content of fucoxanthin but are also notably important as a source of fatty acids, particularly the Omega-3 series of fatty acids such as eicosapentaenoic acid (EPA) and docosahexaenoic acid (DHA), which are highly beneficial to human health [15]. Studies have shown that EPA and DHA are essential fatty acids for aquatic animals, promoting their growth [16]. Currently, fish oil is obtained by extracting fat from deep-sea fish, and it is the main source of essential fatty acids such as EPA and DHA, but with the expansion of aquaculture, the supply of raw materials for fish oil production is insufficient, leading to a continuous rise in fish oil prices [17]. The controlled production conditions of diatoms make them potential alternatives for large-scale stable production of EPA and DHA.

*C. weissflogii* is a marine diatom known for its fast growth and strong adaptability. It can convert light energy into chemical energy stored in organic matter through photosynthesis and fix and utilize carbon through autotrophic methods [18]. Phosphorus is an essential macronutrient for algae growth [3], and both high and low phosphorus concentrations can inhibit the growth and metabolic processes of microalgae. High phosphorus concentrations lead to high intracellular phosphorus accumulation, while low concentrations reduce cell division, affecting protein synthesis, transcription, and carbon cycling [19]. Optimizing phosphorus concentration under controlled conditions may be a key factor in improving production efficiency and commercial value. In environments with limited phosphorus resources, algae may adopt strategies to alter their carbon metabolism pathways in order to adapt to the environmental stress resulting from this condition. For instance, they may enhance their ability to uptake organic carbon sources such as glycerol, or alter the mechanisms of carbon fixation and conversion [20]. Some algae can engage in mixotrophic growth using organic carbon sources like glycerol under phosphorus-deficient conditions, thereby increasing their biomass and product accumulation [21]. The adjustment mechanisms of such metabolic pathways are crucial for algae to maintain growth and metabolic activities under adverse conditions. Furthermore, by precisely regulating the ratio of nitrogen to phosphorus, the growth rate, biomass accumulation, and product formation of algae can be significantly altered. Therefore, in the process of algae cultivation, proper management of the nitrogen-to-phosphorus ratio is a key factor in ensuring efficient growth and effective product accumulation of algae [22,23]. However, the specific mechanism of different phosphorus concentrations in the synthesis of fucoxanthin and the metabolic process of fatty acids is not yet clear. The lack of knowledge in this area hinders the commercial application potential of microalgae.

The effect of phosphorus on *C. weissflogii* is currently unclear. The core objective of this study is to delve into and quantify the effects of different phosphorus concentrations on the growth characteristics of *C. weissflogii*, with a particular focus on their influence on the co-production of fucoxanthin and fatty acid. Solving this problem opens up new avenues for the co-production of these two high-value bioactive compounds and offers theoretical support for the sustainable large-scale utilization of microalgae to produce high-value bioactive compounds.

## 2. Results

### 2.1. The Effect of Different Phosphorus Concentrations on the Growth of C. weissflogii

**Figure 1:**
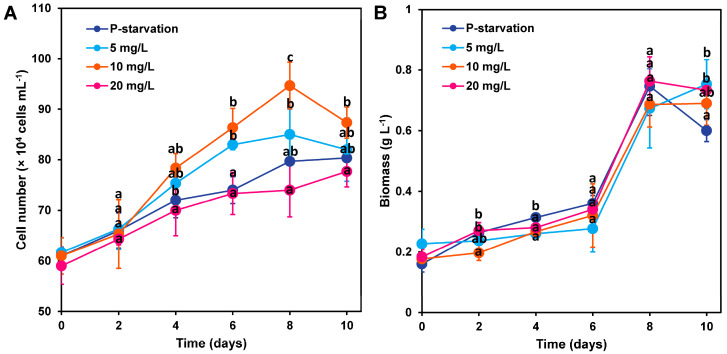
Changes in cell number (**A**) and biomass (**B**) of *C. weissflogii* cultures (mean ± SD, *n* = 3). a, b, c: The same letter indicates that there is no significant difference between treatment groups, and different letters indicate significant difference between treatment groups.

As shown in Figure 1A, phosphorus concentration significantly affects the cell density of *C. weissflogii* (*p* < 0.05). It is noteworthy that in the experimental group with a phosphorus concentration of phosphate starvation, 5 mg L^−1^, and 10 mg L^−1^, the cell density of *C. weissflogii* reached its maximum on the 8th day, showing an initial increase followed by a decrease over the 10-day culture period. However, in the 20 mg L^−1^ phosphorus concentration group, the cell density reached its maximum on the 10th day and continuously increased throughout the 10-day culture period. On the 8th day of the cultivation period, the 10 mg L^−1^ phosphorus concentration group exhibited the highest cell density, peaking at 9.5 × 10^5^ cells mL^−1^, which was 19%, 12%, and 28% higher compared to the phosphate starvation, 5 mg L^−1^, and 20 mg L^−1^ groups, respectively, and this advantage was statistically significant compared to the 20 mg L^−1^ group (*p* < 0.05). It is inferred that a phosphorus concentration of 10 mg L^−1^ is the optimal condition for promoting the growth of *C. weissflogii* cells.

Regarding cell dry weight, as clearly shown in Figure 1B, no significant differences in the dry weight of *C. weissflogii* cells were observed statistically under different phosphorus concentrations (*p* > 0.05). Over the 10-day cultivation period, the cell dry weight in the phosphate starvation and 20 mg L^−1^ groups steadily increased, while the 5 mg L^−1^ and 10 mg L^−1^ groups experienced a trend of first increasing and then decreasing. Specifically, on the 8th day, the cell dry weight in the 20 mg L^−1^ group reached its maximum at 0.76 ± 0.08 g L^−1^, which was approximately 1%, 13%, and 10% higher than the other three groups (phosphate starvation, 5 mg L^−1^, 10 mg L^−1^), although these differences were not statistically significant.

### 2.2. The Effect of Different Phosphorus Concentrations on the Pigment Content of C. weissflogii

**Figure 2:**
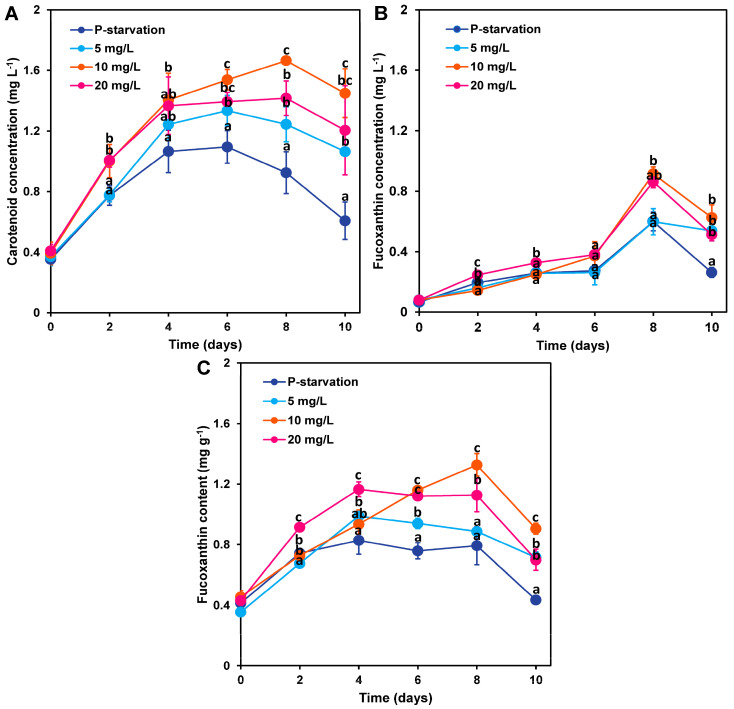
Changes in carotenoid concentration (**A**), fucoxanthin concentration (**B**), and fucoxanthin content (**C**) of *C. weissflogii* cultures (mean ± SD, *n* = 3). a, b, c: The same letter indicates that there is no significant difference between treatment groups, and different letters indicate significant difference between treatment groups.

Phosphorus concentration, as a key variable, significantly affected the accumulation process of carotenoids in *C. weissflogii*, with differences being statistically significant (*p* < 0.05). As shown in Figure 2A, after a 10-day cultivation period, the carotenoid concentration in all phosphorus concentration treatment groups followed a similar trend: initially increasing and then decreasing. It is noteworthy that the experimental group with phosphate starvation lagged significantly in carotenoid accumulation compared to the other groups, exhibiting the lowest level. In contrast, the group with a phosphorus concentration of 10 mg L^−1^ consistently showed higher carotenoid accumulation throughout the cultivation period, maintaining a lead over the other experimental groups. By day 8, this time point became the turning point for carotenoid accumulation, with the 10 mg L^−1^ phosphorus concentration group reaching a peak carotenoid content of 1.67 mg g^−1^. This value was not only higher than any measurement at other time points but also showed a statistically significant difference compared to the other phosphorus concentration treatment groups (*p* < 0.05), further highlighting the important role of 10 mg g^−1^ phosphorus concentration in promoting carotenoid accumulation in *C. weissflogii*.

As shown in Figure 2B, the fucoxanthin concentration in each experimental group increased over time and reached a maximum on day 8 of culture before decreasing rapidly. The maximum value of the 10 mg L^−1^ phosphorus concentration treatment group reached 0.91 mg L^−1^, which was the highest among all experimental groups. The fucoxanthin concentration in the 10 mg L^−1^ group increased by 52%, 52%, and 5.8% compared with the p-starvation, 5 mg L^−1^, and 20 mg L^−1^ groups, respectively.

As shown in Figure 2C, during the 10-day culture period, fucoxanthin content reached its maximum between days 4 and 8, displaying an initial increase (from day 0 to day 4) followed by a decrease (after day 4). The fucoxanthin content in the 10 mg L^−1^ phosphorus concentration group reached its peak on the 8th day, at 1.33 mg g^−1^. Before day 6, the fucoxanthin content in the 20 mg L^−1^ phosphorus concentration group was higher than in the other groups. However, from day 6 to day 10, the fucoxanthin content in the 10 mg L^−1^ phosphorus concentration group surpassed that of the other groups.

**Figure 3:**
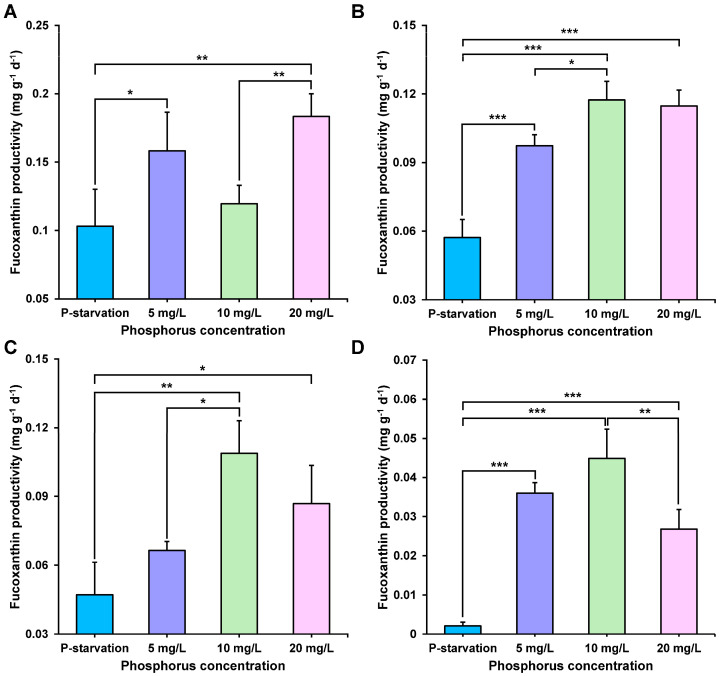
Fucoxanthin productivity on day 4 (**A**), day 6 (**B**), day 8 (**C**), and day 10 (**D**) (means ± SD, *n* = 3) (* *p* < 0.05, ** *p* < 0.01, *** *p* < 0.001).

In the presentation of Figure 3, we compared and analyzed the productivity of fucoxanthin at days 4, 6, 8, and 10 in four different phosphorus concentration treatment groups. The results showed that fucoxanthin in all experimental groups exhibited the highest productivity on the 4th day of cultivation, reaching its peak, and then gradually decreasing with culture time. Further observation of the experimental groups under different phosphorus concentrations revealed that the group with a phosphorus concentration of 10 mg L^−1^ exhibited the highest productivity among all experimental groups on the 6th (Figure 3B), 8th (Figure 3C), and 10th (Figure 3D) day. This finding indicates that setting the phosphorus concentration to 10 mg L^−1^ has a significant positive effect on promoting fucoxanthin production.

### 2.3. Fatty Acid Composition and Concentration of C. weissflosi at Different Phosphorus Concentrations

As shown in Table 1, we conducted a detailed measurement of 25 fatty acids in *C. weissflogii* under different phosphorus concentration conditions. These fatty acids were divided into 13 unsaturated fatty acids (UFAs) and 11 saturated fatty acids (SFAs). It is noteworthy that the analysis results show that the total content of UFAs is significantly higher than the total content of SFAs in all treatment groups, with particularly significant variations in the contents of fatty acids such as C14:0, C16:0, C16:1n7, and C20:5n3. In each experimental group, the total content of UFAs, SFAs, and total fatty acids all increased with rising phosphorus concentrations. The order of fatty acid content from high to low was SFA (43.1–45.1%) > MUFA (monounsaturated fatty acid) (38.2–41.4%) > PUFA (polyunsaturated fatty acid) (13.6–18.6%).

**Figure 4:**
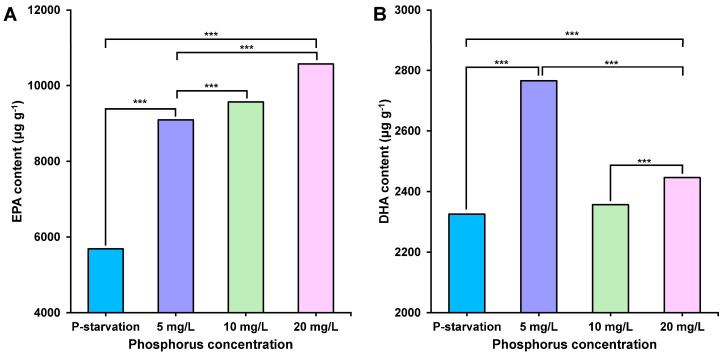
EPA (**A**) and DHA (**B**) content of *C. weissfogii* under different phosphorus concentration culture conditions. Due to the lack of sample quality, three parallel samples were mixed for the determination of EPA and DHA content, and each index was measured three times. (*** *p* < 0.001).

Figure 4 shows the changes in the accumulation of EPA (Figure 4A) and DHA (Figure 4B) in *C. weissflogii* under different phosphorus concentrations. The accumulation of EPA shows a positive correlation with phosphorus concentration, meaning that as the phosphorus concentration increases, the content of EPA steadily rises, peaking at 10.57 mg g^−1^ when the phosphorus concentration is 20 mg L^−1^. The accumulation pattern of DHA exhibits a different trend, namely that its content does not increase monotonically with phosphorus concentration, but instead, it first rises, reaching a maximum of 2.77 mg g^−1^ at 5 mg L^−1^, and then declines.

## 3. Discussion

Phosphorus is an essential trace element that regulates the growth and metabolism of microalgae cells and is a necessary component of DNA, RNA, ATP, and cell membranes [24,25]. This element is the cornerstone of cellular function and its importance permeates a wide range of cellular activities, playing an indispensable role in key processes such as the core mechanisms of energy conversion, intricate signaling networks, the biological construction of complex macromolecules, the source of life through photosynthesis, and the energy-releasing process of respiration [26]. Different concentrations of phosphorus affect the growth of microalgae differently; both excessively high and low phosphorus concentrations can impact algal cell growth and metabolism. When phosphorus is deficient, the energy required for the metabolism of microalgae cells decreases, which is detrimental to cell division. In contrast, at higher phosphorus concentrations, microalgae exhibit phosphorus accumulation, with excess phosphorus stored in vacuoles and chloroplasts, which not only hinders cell growth but also inhibits it [27,28]. Zhang et al. (1999) found that a phosphorus concentration of 4.0–4.5 μmol L^−1^ can promote the growth of microalgae cells, with biomass increasing as the phosphorus concentration rises, though cell biomass exhibits an initial increase followed by a decrease [29]. Other studies have shown that increasing the phosphorus concentration to a certain multiple does not lead to significant changes in cell density or biomass concentration [30]. Mayers et al. (2014) discovered that as phosphorus concentration decreases, the biomass and cell number of *Nannochloropsis* sp. significantly reduce, with low phosphorus concentrations affecting algal cell growth and division [31]. Lopes et al. (2019) reported that as phosphorus concentration increases, the growth rate of algae first rises and then falls, with biomass showing a similar trend, and total lipid content significantly decreases [28]. Within the range of phosphorus concentrations below 10 mg L^−1^, the biomass growth rate of *Tetrahymena obliqua* is positively correlated with the total phosphorus concentration. However, when the phosphorus concentration exceeds 10 mg L^−1^, biomass growth is inhibited [32]. In this experiment, the cell biomass and dry weight of *C. weissflogii* initially increased and then decreased at a phosphorus concentration of 20 mg L^−1^. This is consistent with the experimental results of Zhong Yue et al., which showed that when the phosphorus concentration was 0.072 mmol·L^−1^, the cell biomass and dry weight of *C. weissflogii* reached their maximum values, and the growth trend was also consistent [33]. Therefore, this indicates that while the required concentration range of phosphorus varies among different algae, the growth trends under different phosphorus concentrations are generally consistent across different algae species.

Phosphorus is one of the key elements in pigment synthesis [34]. An appropriate amount of phosphorus can promote the synthesis of algal pigments, increase their content, and thereby improve photosynthesis efficiency and light energy utilization [35]. Production of fucoxanthin is influenced by the catalytic action of a series of enzymes. Phosphorus, as a cofactor of enzymes, has a significant impact on their activity and stability [36]. Zhang et al. found that when culturing *Nannochloropsis*, the proportions of violaxanthin, diadinoxanthin, lutein, and zeaxanthin gradually decreased with lower phosphorus concentrations, with the highest proportions of diadinoxanthin, lutein, violaxanthin, and zeaxanthin occurring at lower phosphorus levels [37]. Studies also indicate that when the phosphorus concentration is 0 in *Dunaliella salina*, the content of β-carotene and chlorophyll is low. However, when the phosphorus concentration increases to 0.2 mmol L^−1^, the content of β-carotene and chlorophyll reaches a higher level, with pigment content increasing as phosphorus concentration rises [38]. Chen et al. (2019) discovered that increasing phosphorus concentration is beneficial for enhancing the content of protein, phycocyanin, and photosynthetic pigments in *Spirulina platensis* [34]. These contents reach their maximum when the phosphorus concentration is 0.02 g L^−1^, but further increases in phosphorus concentration cause a slight decrease in these contents [39]. The effect of phosphorus on photosynthetic pigment content may be related to changes in proteins (enzymes), thus showing a similar overall trend [40]. Additionally, changes in photosynthetic pigments can influence protein, polysaccharide, and energy metabolism processes, although other physiological processes might also be involved [41]. Salman et al. found that with increasing phosphorus concentration, the contents of protein, phycocyanin, chlorophyll, and carotenoids in *Spirulina platensis* significantly increased [42]. In this experiment, we also observed that as the phosphorus concentration increased, the contents of carotenoids and fucoxanthin first increased and then decreased, reaching their maximum at phosphorus concentrations of 10–20 mg L^−1^. This is also consistent with previous experimental results which indicate that 0.072 mmol·L^−1^ potassium dihydrogen phosphate is conducive to the co-production of fucoxanthin [33,43]. At the same time, this also confirms that different algae species have varying requirements for phosphorus concentrations in pigment accumulation conditions.

Phosphorus concentration not only affects the growth of microalgae but also influences the accumulation of bioactive substances in microalgae [25]. Fatty acids are considered essential for the survival of many marine animals in their early life stages [44,45], and EPA and DHA, as representatives of ω-3 unsaturated fatty acids, are abundant in diatoms and have significant nutritional value [46,47]. Changes in phosphorus concentration can alter the fatty acid composition within microalgae cells [48,49]. Under phosphorus starvation conditions, intracellular lipid content increases, and due to phosphorus deficiency, there is often a conversion from phospholipids to non-phospholipids [50]. Phosphorus, serving as a cofactor for enzymes and a constituent of energy molecules, plays a crucial role in the synthesis process of fatty acids [51]. Similar to the synthesis of fucoxanthin, the synthesis of fatty acids also requires a substantial energy supply. A deficiency in phosphorus can lead to inadequate energy provision, thereby affecting the synthesis rate of fatty acids [52]. Furthermore, phosphorus also participates in metabolic regulation processes by influencing the expression of relevant genes and the activity of enzymes, thereby regulating the synthesis and catabolism of fatty acids [53]. Mandal et al. found that the lipid content in *Scenedesmus obliquus* significantly increased under phosphorus-deficient conditions, rising from 12.7% to 30%. The total fatty acid content also increased, with a rise in monounsaturated fatty acids and a decrease in polyunsaturated fatty acids, indicating an inverse trend in lipid content with increasing phosphorus concentration [54]. Roopnarain et al. (2014) showed that when the phosphorus content was 0%, 1%, 5%, and 12.5%, lipid accumulation reached its maximum, and phosphorus content in the medium below 25% triggered a cellular response to phosphorus starvation [55]. Shi et al. (2020) found that the accumulation rate of most fatty acids under phosphorus-deficient conditions was significantly higher than under normal conditions, with different fatty acids being affected to varying degrees as phosphorus concentration decreased. Changes in phosphorus concentration also impact the levels of EPA and DHA [25]. Yongmanitchai et al. (1991) observed that by doubling the phosphorus concentration in the medium of *Phaeodactylum tricornutum* (from 0.05 g L^−1^ to 0.1 g L^−1^), the EPA content increased from 20.1% to 27.1% of total fatty acids, while the SFA proportion initially decreased and then increased, and the UFA proportion initially increased and then decreased as phosphorus concentration decreased [56]. An optimal phosphorus concentration promotes the synthesis of EPA and PUFA [57]. Increasing phosphorus concentration may enhance the photosynthetic activity of *C. weissflogii*, improve its light energy conversion efficiency, and subsequently promote the growth of algal biomass and product accumulation [58,59]. The results of this study are consistent with previous observations, specifically manifested as follows: when the phosphorus concentration is optimized to 20 mg L^−1^, not only is the total amount of fatty acids maximized, but the content of EPA also peaks; on the other hand, when the phosphorus concentration is set to 5 mg L^−1^, the production of DHA reaches its highest level. This finding once again confirms the critical impact of phosphorus concentration on the accumulation of fatty acids, especially specific types of fatty acids. 

## 4. Materials and Methods

### 4.1. C. weissflogii Strain and Culture Conditions

*C. weissflogii* was provided by the Algal Resource Development and Aquatic Environmental Ecological Restoration Laboratory at Guangdong Ocean University (Zhanjiang, China) [57]. 

The strain was grown photosynthetically in a 5 L flask for 24 h under a constant temperature of 25 °C and a constant light intensity of 30 ± 2 μmol m^−2^ s^−1^, with continuous aeration to promote growth. The modified F/2 medium (see Tables S1 and S2) was added to pre-filtered seawater for the cultivation process.

### 4.2. Experimental Setup

Based on previous research, in this study, potassium dihydrogen phosphate (KH_2_PO_4_) was used as the phosphorus nutrient, with different phosphorus concentration gradients designed at phosphate starvation, 5 mg L^−1^, 10 mg L^−1^, and 20 mg L^−1^ [18,33]. The experiment was conducted in a glass cylindrical photobioreactor (inner diameter of 5 cm, height of 60 cm), with an initial culture volume of 700 mL and containing microorganisms with an initial cell concentration of approximately 6 × 10^5^ cells mL^−1^. The cultivation conditions were as follows: temperature of 25 ± 2 °C, light intensity of 30 ± 2 μmol m^−2^ s^−1^, salinity of 25 ± 2, pH of 8.0 ± 0.2, and aeration rate (air) of 0.4 L min^−1^. The side lighting was provided by T8 LED white light tubes, set for continuous illumination without a dark period, and the cultivation period was 10 days. Each treatment was repeated three times to ensure the reproducibility of the experiment.

### 4.3. Analytical Methods

A 0.1 mL sample was taken from the experimental samples and observed under an Olympus BX53 light microscope at a magnification of 10 × 40 using a hemocytometer (25 mm × 16 mm).

The biomass was measured by using a dry weight method according to Rui et al [57]. To measure the dry weight (M), a 10 mL algal sample was filtered using a pre-weighed (M_1_) cellulose acetate filter membrane (pore size 1 μm). The filter membrane was washed twice with distilled water, and then the samples were transferred to an oven set at 80 °C for continuous drying until their mass reached a constant state. During this process, we recorded detailed data on the total mass of the dried filter membranes as M_2_. The dry weight of the algal cells was calculated using Equation (1):

(1) $$M=(M_{2}-M_{1})/10$$

Using ethanol as a solvent, the content of carotenoids in the sample was determined by using an extraction method [18,60]. A 10 mL algal sample was centrifuged at 5000 r min^−1^ for 10 min, and the algal cells were collected. The supernatant was discarded, and 10 mL of 95% ethanol was added. After 24 h of dark treatment, the sample was centrifuged again at 5000 r min^−1^ for 10 min, and the supernatant was collected. The optical density of the supernatant was measured at wavelengths of 480 nm, 510 nm, and 750 nm using a spectrophotometer. The carotenoid content was calculated using Equation (2):

(2) $$\rho (Carotenoids)=7.6\times [(D_{480}-D_{750})-1.49\times (D_{510}-D_{750})]$$

The fucoxanthin content in algal cells was determined using the organic solvent extraction method [61]. At 4 °C, an 80 mL algal sample was centrifuged at 5000 r min^−1^ for 10 min. The supernatant was discarded, and the algal cells were freeze-dried for 2 days. The freeze-dried algal powder was then added to anhydrous ethanol (at a ratio of 1 g:40 mL) and extracted twice in the dark, each time for 1 hour. After extraction, the algal solution was centrifuged again at 5000 r min^−1^ for 10 min, and the supernatant was collected. The optical density of the supernatant at 445 nm (D_445_) was measured using a UV spectrophotometer. The fucoxanthin content was calculated using Equation (3):

(3) $$C=(1000\times D_{445}\times N\times V)/(A^{\prime }\times M\times 100)$$

In the formula, C represents the fucoxanthin content in mg g^−1^; N is the dilution factor; V is the volume of the crude extract; A′ is the theoretical absorbance value of a 1% solute in a 1 cm cuvette, which is 1600; M is the mass of the sample being tested.

The fucoxanthin concentration was calculated using Equation (4):

(4) FC=M×C

In the formula, FC represents the fucoxanthin concentration in mg L^−1^; M is the dry weight in g L^−1^; C is the fucoxanthin content in mg g^−1^.

According to GB 5009.168-2016 [62], the fatty acid composition of the algal samples was determined using gas chromatography. The gas chromatography conditions were as follows: column (DB-WAX): 30 m × 0.32 mm × 0.50 μm; carrier gas: nitrogen, with a constant flow of 1.5 mL min^−1^; injection port: a split ratio of 20:1, a temperature of 240 °C, and an injection volume of 1 μL; detector temperature: 240 °C; oven temperature program: hold at 80 °C for 3.0 min, then increase to 120 °C at 5.0 °C min^−1^ and hold for 5.0 min, then increase to 180 °C at 2.0 °C min^−1^ and hold for 50 min, and finally increase to 240 °C at 1.0 °C min^−1^ and hold for 60 min. The 37-Component FAME standard mixture (p/n CDAA-252795-MIX-1 mL) and single standards of C20:5n3 (p/n CDAA-253209M-10 mg) and C22:6n3 (p/n CDAA-253228M-10 mg) were purchased from ANPEL Scientific Instrument Co. Ltd. (Shanghai, China). The concentration of each component in the mixture was 200 to 400 mg mL^−1^. Data processing was performed as follows: fatty acid components were identified by comparing retention times with fatty acid methyl ester standards and quantified using the area normalization method.

Data were processed and charts were created using Excel 2019. One-way ANOVA with Duncan’s tests (post hoc) were performed using SPSS 26.0 to analyze differences in algal cell density, dry weight, carotenoid content, and fucoxanthin content. The significance level was set as *p* < 0.05, and the results are expressed as the mean ± SD. After one-way ANOVA tests, LSD (Least Significant Difference) tests were performed for the 4 treatment groups to analyze fucoxanthin productivity, EPA content, and DHA content. The significance level was set as * *p* < 0.05, ** *p* < 0.01, and *** *p* < 0.001. Sensitivity analysis was performed for fatty acid content due to the insufficient quality of the parallel samples.

## 5. Conclusions

This study explored the regulatory effect of phosphorus concentration in the medium on the growth performance and accumulation of bioactive substances of *C. weissflogii*. The study revealed that by regulating the phosphorus concentration, the accumulation of key metabolites in *C. weissflogii* can be significantly optimized. When the phosphorus concentration was set at 5 mg L^−1^, this condition was most suitable for the expansion of algal cell density and the maximization of DHA content; when the phosphorus concentration was increased to 10 mg L^−1^, it promoted the significant accumulation of pigment substances such as carotenoids and fucoxanthin; further, adjusting the phosphorus concentration to 20 mg L^−1^ became the optimal condition for the EPA content and total fatty acid content to reach their peak. At a concentration of 10 mg L^−1^, *C. weissflogii* exhibited rapid growth, ease of cultivation, and the ability to accumulate large amounts of fucoxanthin in a relatively short period of time, making it suitable for commercial-scale production. Although *C. weissflogii* itself may not be a major producer of EPA/DHA, marine microalgae as a group possess significant advantages in EPA/DHA production, and their research and development value cannot be overlooked. These research achievements have laid a solid foundation for advancements in large-scale microalgae cultivation technology, particularly paving the way for efficient and targeted production of high-value bioactive compounds. This study has deepened our understanding of microalgae biotechnology and highlighted the potential of microalgae as important compounds for promoting the sustainable health of aquatic organisms and humans. Additionally, this research has provided valuable experimental guidance and support for large-scale industrial production, potentially having a profound impact on fields such as nutritional supplements, aquaculture, and the development of functional foods and health supplements.

## Figures and Tables

**Table 1:** Fatty acid composition and concentrations (μg g^−1^) of C. weissflogii under different phosphorus concentrations.

Test Items	Phosphorus Concentration			
	P starvation	5 mg L^−1^	10 mg L^−1^	20 mg L^−1^
C8:0	97.77	Not detected	92.59	133.93
C10:0	363.62	406.17	170.17	340.24
C11:0	117.72	211.36	340.30	238.79
C12:0	154.71	312.40	153.32	123.18
C14:0	6912.32	8406.19	9721.57	10,267.69
C14:1n5	167.34	96.88	253.04	268.99
C15:0	1078.67	1561.63	1602.87	1993.21
C16:0	22,499.15	25,320.68	25,763.80	28,738.26
C16:1n7	29,619.13	32,494.87	36,096.57	38,151.92
C18:0	445.70	658.26	644.12	660.41
C18:1n9c	749.10	1107.47	560.54	719.00
C18:2n6c	202.22	548.19	550.91	615.57
C18:3n6	160.64	223.71	224.99	274.44
C18:3n3	871.77	191.36	255.70	246.65
C20:0	12.16	104.16	15.38	141.44
C20:2	36.27	20.66	27.06	21.45
C20:3n6	37.97	30.80	22.13	16.00
C20:4n6	350.77	484.69	522.13	927.00
C20:3n3	352.00	256.66	212.38	292.47
EPA	5687.71	9092.24	9571.54	10,574.29
C22:0	174.54	97.44	189.14	68.14
C22:2n6	Not detected	Not detected	2200.32	3651.27
DHA	2326.10	2765.83	2356.87	2445.98
C23:0	451.36	838.18	487.82	793.05
C24:0	847.48	1008.07	925.00	784.47
SFA (%)	45.0	45.1	43.1	43.2
MUFA (%)	41.4	39.1	39.7	38.2
PUFA (%)	13.6	15.8	17.2	18.6
UFA (%)	55.0	54.9	56.9	56.8
FA	73,716.22	86,237.90	92,960.26	102,487.84

## Data Availability

The original data presented in this study are included in the article/Supplementary Materials; further inquiries can be directed to the corresponding author.

## Supplementary Materials

The following supporting information can be downloaded at https://www.mdpi.com/article/10.3390/md22120541/s1: Table S1: f/2 medium; Table S2: f/2 trace metal solution.

## References

[1] Sigrún H.J. (2019). Fatty Acid Profiles and Production in Marine Phytoplankton. Mar. Drugs.

[2] Zhou L., Li K., Duan X., Hill D., Barrow C., Dunshea F., Martin G., Suleria H. (2022). Bioactive Compounds in Microalgae and Their Potential Health Benefits. Food Biosci..

[3] Khan M.I., Shin J.H., Kim J.D. (2018). The Promising Future of Microalgae: Current Status, Challenges, And Optimization of a Sustainable and Renewable Industry for Biofuels, Feed, and Other Products. Microb. Cell Fact..

[4] Ampofo J., Abbey L. (2022). Microalgae: Bioactive Composition, Health Benefits, Safety and Prospects as Potential High-Value Ingredients for The Functional Food Industry. Foods.

[5] Jing F., Yifu G., Heyu W. (2023). Research on the Effects of Culture Factors on the Growth and Fucoxanthin of *Phaeodactylum tricornutum*. Ind. Microbiol..

[6] Dolganyuk V., Belova D., Babich O., Prosekov A., Ivanova S., Katserov D., Patyukov N., Sukhikh S. (2020). Microalgae: A Promising Source of Valuable Bioproducts. Biomolecules.

[7] Wang W., Yu L.J., Xu C., Tomizaki T., Zhao S., Umena Y., Chen X., Qin X., Xin Y., Suga M. (2019). Structural Basis for Blue-Green Light Harvesting and Energy Dissipation in Diatoms. Science.

[8] Guo Y.F., Wang S.J., Chen B.F., Wang S., Men J., Qiao Z.X., Luo H.-Z., Jin H. (2024). Optimization of Conditions for Heterotrophic-Light Coupling High-Density Cultivation of *Poterioochromonas malhamensis* for Fucoxanthin Production. Acta Hydrobiol. Sin..

[9] Li Y., Sun H., Wu T., Fu Y., He Y., Mao X., Chen F. (2019). Storage Carbon Metabolism of *Isochrysis zhangjiangensis* Under Different Light Intensities and Its Application for Co-Production of Fucoxanthin and Stearidonic Acid. Bioresour. Technol..

[10] Wang L., Fan Y., Parson R.L., Hu G.R., Zhang P.Y., Li F.L. (2018). A Rapid Method for the Determination of Fucoxanthin in Diatom. Mar. Drugs.

[11] Maoka T., Kaczor A., Baranska M. (2016). Structural Studies of Carotenoids in Plants, Animals, and Food Products. Carotenoids: Nutrition, Analysis and Technology.

[12] Borowitzka M.A. (2013). High-Value Products from Microalgae—Their Development and Commercialisation. J. Appl. Phycol..

[13] Wan X., Zhou X.R., Moncalian G., Su L., Chen W.C., Zhu H.Z., Chen D., Gong Y.-M., Huang F.-H., Deng Q.-C. (2021). Reprogramming Microorganisms for The Biosynthesis of Astaxanthin via Metabolic Engineering. Prog. Lipid Res..

[14] Liaqat F., Khazi M.I., Bahadar A., He L., Aslam A., Liaquat R., Agathos S., Li J. (2023). Mixotrophic Cultivation of Microalgae for Carotenoid Production. Rev. Aquac..

[15] Zhan J.S., Zhan K., Zhao G.Q. (2013). DHA: Extraction Techniques and Application in Animal Husbandry. Chin. J. Anim. Nutr..

[16] Jiang D. (2017). Effect of Different DHA Contents in Feed on Growth, Digestion and Physiological and Biochemical Index of *Pelodiscus sinensis*. Master’s Thesis.

[17] Meng W., Wang Q., Mou H., Gong Y., Shen Y.W., Sun Y.X., Mai K.S. (2022). Effects of Palmitic acid/(EPA+DHA) Ratios on Anti-oxidative Capacity and Muscle Quality of Large Yellow Croaker *Larimichthys crocea*. Prog. Fish. Sci..

[18] Rui X., Amenorfenyo D.K., Peng K., Li H.M., Wang L.F., Huang X., Li C., Li F. (2023). Effects of Different Nitrogen Concentrations on Co-Production of Fucoxanthin and Fatty Acids in *Conticribra weissflogii*. Mar. Drugs.

[19] Shao Q. (2023). Construction and Application of a Phosphorus Metabolism model for Microalgae Growth. Master’s Thesis.

[20] Nur M.M.A., Muizelaar W., Boelen P., Buma A.G.J. (2019). Environmental and nutrient conditions influence fucoxanthin productivity of the marine diatom *Phaeodactylum tricornutum* grown on palm oil mill effluent. J. Appl. Phycol..

[21] Nur M.M.A. (2021). Co-production of fucoxanthin and lipid from Indonesian diatom and green algae growing on palm oil mill effluent under mixotrophic condition. Biocatal. Agric. Biotechnol..

[22] Muhamad M.A.N., Fonda M.L., Ana N.L.D.P., Titi T.A., Harsa P., Ira N.D. (2024). Enhancing fucoxanthin production in *Chaetoceros calcitrans* under mixotrophic condition and LED wavelength shift strategy. Biocatal. Agric. Biotechnol..

[23] Muhamad M.A.N., Ira N.D., Nugroho A.S., Agusta S.P., Hadiyanto (2023). Co-cultivation of *Chaetoceros calcitrans* and *Arthrospira platensis* growing on palm oil mill effluent under outdoor condition to produce fucoxanthin and c-phycocyanin. Biocatal. Agric. Biotechnol..

[24] Wang B., Li Y.Q., Wu N., Lan C.Q. (2008). CO_2_ Bio-Mitigation Using Microalgae. Appl. Microbiol. Biotechnol..

[25] Shi Y., Liu M.J., Ding W., Liu J. (2020). Novel Insights into Phosphorus Deprivation Boosted Lipid Synthesis in the Marine Alga *Nannochloropsis Oceanica* Without Compromising Biomass Production. J. Agric. Food Chem..

[26] Raghothama K.G. (2000). Phosphate transport and signaling. Curr. Opin. Plant Biol..

[27] Sun S. (2009). Selection of Rich Lipids-Microalgae and the Research on the Factors Affecting the Lipid Content and Lipid Composition of Microalgae. Master’s Thesis.

[28] Lopes R.G., Cella H., Mattos J.J., Marque M.R.F., Soares A.T., Filho N.R.A., Derner R.B., Rörig L.R. (2019). Effect of Phosphorus and Growth Phases on The Transcription Levels of EPA Biosynthesis Genes in The Diatom *Phaeodactylum tricornutum*. Braz. J. Bot..

[29] Wu Y.H., Yu Y., Li X., Hu H.Y., Su Z.F. (2012). Biomass Production of a Scenedesmus sp. Under Phosphorous-Starvation Cultivation Condition. Bioresour. Technol..

[30] Zhang Y.H., Lian Y.W. (1999). Effects of Phosphorus on the Growth of the Red Tide Organism *Alexandrium tamarense*. Mar. Environ. Sci..

[31] Mayers J.J., Flynn K.J., Shields R.J. (2014). Influence of the N:P Supply Ratio on Biomass Productivity and Time-Resolved Changes in Elemental and Bulk Biochemical Composition of *Nannochloropsis* sp.. Bioresour. Technol..

[32] Huang Y.F. (2024). Physiological Characteristics and Pollutant Purification Effect of *Tetrahymena obliqua* Cultivated with Different Phosphorus Concentrations. Master’s Thesis.

[33] Zhong Y., Su Y.C., Chen Y.Q., Chen J.N. (2022). The effects of different nitrogen and phosphorus nutrients on the growth and fucoxanthin accumulation in *Thalassiosira weissflogii*. J. Fish. Res..

[34] Chen H., Yang B.J., Li T., Wu H.L., Wu H.B., Xiang W.Z. (2019). Effects of Phosphorus Concentrations on Growth and Metabolism of Seawater *Spirulina platensis*. Biotechnol. Bull..

[35] Yuan X., Liang L., Liu K., Xie L.J., Huang L.Q., He W.J., Chen Y., Xue T. (2019). Spent Yeast as An Efficient Medium Supplement for Fucoxanthin and Eicosapentaenoic Acid (EPA) Production by *Phaeodactylum tricornutum*. J. Appl. Phycol..

[36] Zhang Y.P., Xie Q.L., Fang H., Sun J.P., Hong Z., Yi R.Z., Wu H. (2013). Research progress of Fucoxanthin. Chin. J. Drugs.

[37] Zhang Y.B., Tian J.J., Ye L.Z., Ye Z.W., Zhang L., Xu J.L. (2022). Effects of Several Environmental Factors on Nutrient Accumulations of *Nannochloropsis oceanica*. J. Nucl. Agric. Sci..

[38] Sun H., Xu W.H., Lei G.P., Deng T.T., Yang W.G., Liu Y.K., Cao Y., Bai L.H. (2005). The Effect of Nitrogen, Phosphorus, and Sulfur on the Pigments Accumulation of *Dunaliella salina*. J. Sichuan Univ. Nat. Sci. Ed..

[39] Xu K. (2018). Research on the Growth and Photosynthesis of *Chlorella vulgaris* under Different Nitrogen and Phosphorus Concentrations. Master’s Thesis.

[40] Geider R., Roche J.L. (2002). Redfield Revisited: Variability of C:N:P in Marine Microalgae and Its Biochemical Basis. Eur. J. Phycol..

[41] Khoeyi Z.A., Seyfabadi J., Ramezanpour Z. (2012). Effect of Light Intensity and Photoperiod on Biomass and Fatty Acid Composition of the Microalgae, *Chlorella vulgaris*. Aquac. Int..

[42] Mansour M.P., Frampton D.M.F., Nichols P.D., Volkman J.K., Blackburn S.I. (2005). Lipid and fatty acid yield of nine stationary-phase microalgae: Applications and unusual C_24_-C_28_ polyunsaturated fatty acids. J. Appl. Phycol..

[43] Sui J.K., Wang H., Liu T.Z. (2019). Research progress of the characteristics and biosynthesis of diatom fucoxanthin. Mar. Sci..

[44] Sandnes J.M., Källqvist T., Wenner D., Gislerød H.R. (2005). Combined Influence of Light and Temperature on Growth Rates of *Nannochloropsis oceanica*: Linking Cellular Responses to Large-Scale Biomass Production. J. Appl. Phycol..

[45] Spolaore P., Claire J.C., Duran E., Isambert A. (2006). Commercial Applications of Microalgae. J. Biosci. Bioeng..

[46] Patil V., Källqvist T., Olsen E., Vogt G., Gislerød H.R. (2007). Fatty Acid Composition of 12 Microalgae for Possible Use in Aquaculture Feed. Aquac. Int..

[47] Liang K.H., Zhang Q.H., Gu M., Cong W. (2013). Effect of Phosphorus on Lipid Accumulation in Freshwater Microalga *Chlorella* sp.. J. Appl. Phycol..

[48] Minhas A.K., Peter H., Barrow C.J., Adholeya A. (2016). A Review on the Assessment of Stress Conditions for Simultaneous Production of Microalgal Lipids and Carotenoids. Front. Microbiol..

[49] Zhang X., Liu J.H. (2019). Advances on Lipid Synthetic Pathway and Regulation Mechanism of Microalgae. Genom. Appl. Biol..

[50] Michelon W., Silva M.L.B.D., Mezzari M.P., Pirolli M., Prandini J.M., Soares H.M. (2015). Effects of Nitrogen and Phosphorus on Biochemical Composition of Microalgae Polyculture Harvested from Phycoremediation of Piggery Wastewater Digestate. Appl. Biochem. Biotechnol..

[51] Yaakob M.A., Mohamed R.M.S.R., Al-Gheethi A., Gokare R.A., Ambati R.R. (2021). Influence of Nitrogen and Phosphorus on Microalgal Growth, Biomass, Lipid, and Fatty Acid Production: An Overview. Cells.

[52] Wu L.H., Li T., Wang G.H., Dai S.K., He H., Xiang W.Z. (2016). A comparative analysis of fatty acid composition and fucoxanthin content in six *Phaeodactylum tricornutum* strains from different origins. Chin. J. Oceanol. Limnol..

[53] Courbebaisse M., Souberbielle J.C. (2011). Phosphocalcic metabolism: Regulation and explorations. Nephrol. Ther..

[54] Roopnarain A., Gray V.M., Sym S.D. (2014). Phosphorus Limitation and Starvation Effects on Cell Growth and Lipid Accumulation in *Isochrysis galbana* U4 for Biodiesel Production. Bioresour. Technol..

[55] Yongmanitchai W., Ward P. (1991). Growth of and Omega-3 Fatty Acid Production by *Conticribra weissflogii* under Different Culture Conditions. Appl. Environ. Microbiol..

[56] Liang J.J., Jiang X.M., Ye L., Han Q.X. (2016). Effects of Nitrogen, Phosphorus, and Iron on the Growth, Total Lipid Content, and Fatty Acid Composition of *Phaeodactylum tricornutum* Mutant Strain. Chin. J. Ecol..

[57] Li F., Rui X.Y., Amenorfenyo D.K., Pan Y., Huang X.H., Li C.L. (2023). Effects of Temperature, Light and Salt on the Production of Fucoxanthin from *Conticribra weissflogii*. Mar. Drugs.

[58] Canfield D.E., Bjerrum C.J., Zhang S., Wang H., Wang X. (2020). The modern phosphorus cycle informs interpretations of Mesoproterozoic Era phosphorus dynamics. Earth Sci. Rev..

[59] Feng W.Y., Wang T.K., Zhu Y.R., Sun F.H., Giesy J.P., Wu F.C. (2023). Chemical composition, sources, and ecological effect of organic phosphorus in water ecosystems: A review. Carbon Res..

[60] Parsons T.R., Timothy R.P., Maita Y., Lalli C.M. (1984). Determination of Chlorophylls and Total Carotenoids: Spectrophotometric Method. A Manual of Chemical & Biological Methods for Seawater Analysis.

[61] Xu R.J., Gong Y.F., Chen W.T., Li S.R., Chen R.Y., Zheng X.H. (2019). Effects of LED Monochromatic Light Quality of Different Colors on Fucoxanthin Content and Expression Levels of Related Genes in *Phaeodactylum tricornutum*. Acta Opt. Sin..

[62] (2023). National Food Safety Standard—Determination of Fatty Acid in Foods.

