# Supplementary material for: The Effect of Phosphorus Concentration on the Co-Production of Fucoxanthin and Fatty Acids in Conticribra weissflogii
Source: Mar Drugs. 2024 Nov 30;22(12):541. doi: 10.3390/md22120541 (PMC11678120; doi:10.3390/md22120541)
Supplement: Supplementary file 1 [file marinedrugs-22-00541-s001.zip › marinedrugs-3321304-supplementary.pdf]

Supplementary Materials

To prepare, begin with 950 mL of filtered natural seawater and add the following components. Bring the final volume to 1 liter with filtered natural seawater. Autoclave.

Table S1 f/2 Medium

Component	Stock Solution	Quantity	Molar Concentration in Final Medium
$NaNO_3$	75 g L^{-1} dH_2O	1 mL	8.82×10^{-4} M
$NaH_2PO4\cdot H_2O$	5 g L^{-1} dH_2O	1 mL	3.62×10^{-5} M
$Na_2SiO_3\cdot 9H_2O$	30 g L^{-1} dH_2O	1 mL	1.06×10^{-4} M
trace metal solution	(see recipe below)	1 mL	---

To prepare, begin with 950 mL of dH_2O, add the components and bring final volume to 1 liter with dH_2O. Autoclave. Note that the original medium (Guillard and Ryther 1962) used ferric sequestrene; we have substituted $Na_2EDTA\cdot 2H_2O$ and $FeCl_3\cdot 6H_2O$.

Table S2 f/2 Trace Metal Solution

Component	Primary Stock Solution	Quantity	Molar Concentration in Final Medium
$FeCl_3\cdot 6H_2O$	---	3.15 g	1.17×10^{-5} M
$Na_2EDTA\cdot 2H_2O$	---	4.36 g	1.17×10^{-5} M
$CuSO_4\cdot 5H_2O$	9.8 g L^{-1} dH_2O	1 mL	3.93×10^{-8} M
$Na_2MoO_4\cdot 2H_2O$	6.3 g L^{-1} dH_2O	1 mL	2.60×10^{-8} M
$ZnSO_4\cdot 7H_2O$	22.0 g L^{-1} dH_2O	1 mL	7.65×10^{-8} M
$CoCl_2\cdot 6H_2O$	10.0 g L^{-1} dH_2O	1 mL	4.20×10^{-8} M
$MnCl_2\cdot 4H_2O$	180.0 g L^{-1} dH_2O	1 mL	9.10×10^{-7} M
